# Skeletal muscle oxygenation during cardiopulmonary resuscitation as a predictor of return of spontaneous circulation: a pilot study

**DOI:** 10.1186/s40001-023-01393-z

**Published:** 2023-10-11

**Authors:** Miha Košir, Hugon Možina, Matej Podbregar

**Affiliations:** 1 Faculty of Medicine, University of Ljubljana, Vrazov Trg 2, 1000 Ljubljana, Slovenia; 2 Unit SNMP, Community Health Centre Ljubljana, Bohoričeva Ulica 4, 1000 Ljubljana, Slovenia; 3 Emergency Department, University Medical Center Ljubljana, Zaloška Cesta 4, 1000 Ljubljana, Slovenia; 4 Department for Internal Intensive Care, General Hospital Celje, Oblakova Ulica 5, 3000 Celje, Slovenia

**Keywords:** Near-infrared spectroscopy, Tissue oxygenation, Skeletal muscle, Brain, cardiac arrest, Return of spontaneous circulation

## Abstract

**Background:**

Near-infrared spectroscopy (NIRS) provides regional tissue oxygenation (rSO_2_) even in pulseless states, such as out-of-hospital cardiac arrest (OHCA). Brain rSO_2_ seems to be important predictor of return of spontaneous circulation (ROSC) during cardiopulmonary resuscitation (CPR). Aim of our study was to explore feasibility for monitoring and detecting changes of skeletal muscle rSO_2_ during resuscitation.

**Methods:**

Skeletal muscle and brain rSO_2_ were measured by NIRS (SenSmart Model X-100, Nonin, USA) during CPR in adult patient with OHCA. Start (basal) rSO_2_, maximal during CPR (maximal) and difference between maximal–minimal rSO_2_ (delta-rSO_2_), were recorded. Patients were divided into ROSC and NO-ROSC group.

**Results:**

20 patients [age: 66.0ys (60.5–79.5), 65% male] with OHCA [50% witnessed, 70% BLS, time to ALS 13.5 min (11.0–19.0)] were finally analyzed. ROSC was confirmed in 5 (25%) patients. Basal and maximal skeletal muscle rSO_2_ were higher in ROSC compared to NO-ROSC group [49.0% (39.7–53.7) vs. 15.0% (12.0–25.2), *P = * 0.006; 76.0% (52.7–80.5) vs. 34.0% (18.0–49.5), *P = * 0.005, respectively]. There was non-linear cubic relationship between time of collapse and basal skeletal muscle rSO_2_ in witnessed OHCA and without BLS (F-ratio = 9.7713, *P = * 0.0261). There was correlation between maximal skeletal muscle and brain rSO_2_ (*n = *18, rho: 0.578, *P =  * 0.0121).

**Conclusions:**

Recording of skeletal muscle rSO_2_ during CPR in patients with OHCA is feasible. Basal and maximal skeletal muscle rSO_2_ were higher in ROSC compared to NO-ROSC group.

*Clinical trial registration number* ClinicalTrials.gov, NCT04058925, registered on: 16th August 2019. URL of trial registry record: https://www.clinicaltrials.gov/ct2/show/NCT04058925?titles=Tissue+Oxygenation+During+Cardiopulmonary+Resuscitation+as+a+Predictor+of+Return+of+Spontaneous+Circulation&draw=2&rank=1.

## Background

Out of hospital cardiac arrest (OHCA) is a major cause of morbidity and mortality around the world [1]. Despite the progress in medicine, new devices and research, there was no great breakthrough in recent years [2]. There are numerous factors that have influence on the outcome of cardiopulmonary resuscitation (CPR) in OHCA. It is usually impossible to predict weather the resuscitation will be successful or not, especially in early stages of cardiac arrest [3].

Near-infrared spectroscopy (NIRS) is a noninvasive optical technique that uses light in near-infrared spectrum of electromagnetic wave (700–1300 nm) [4, 5]. NIRS can be used to assess tissue oxygenation, oxygen consumption and blood flow in various tissues, including brain and skeletal muscle [6–8]. NIRS has an advantage over pulsatile oximetry, because it can be used in situations, where there is no blood flow [9]. With NIRS we measure oxygen saturation in all vessels that are smaller in diameter than 1 mm (arterioles, capillaries, venules) [4, 10]. Most of the signal comes from capillaries, because they represent majority of vessels in the tissue [6].

During cardiac arrest and during CPR the blood flow through the brain is absent or significantly reduced. Brain injury is major cause for neurologic disability after successful resuscitation [4, 7, 10]. With the placement of NIRS probes on the forehead region, regional tissue oxygen saturation (rSO_2_) in superficial areas of frontal brain lobes is measured. Current data confirms that increase of brain rSO_2_ is associated with higher probability of return of spontaneous circulation (ROSC) [4, 7, 9–12]. Different values of basal brain rSO_2_ or different values of brain rSO_2_ increase were associated with higher probability of ROSC. Genbrugge et al. reported that absolute increase of rSO_2_ for 15% or more was associated with ROSC. They also noticed that increase in rSO_2_ beyond 1 min after initiation of rSO_2_ measurement was associated with more favorable long-term neurologic outcome [13]. Parnia et al. have shown that all patients with ROSC had mean brain rSO_2_ of 35% or higher. A rise of brain rSO_2_ from baseline was associated with ROSC and values remaining below 30% most of the period of CPR predicted that ROSC will not be achieved [12]. Recent meta-analysis has confirmed prognostic value of brain tissue oxygenation [14].

During cardiac arrest there is no blood flow, the consequence is lower oxygen values in all tissues [15, 16]. Skeletal muscles are not part of vital organs and flow through them is decreased in critical states. In critically ill we currently monitor skeletal muscle rSO_2_ in patients with shock or in patients on different types of circulatory mechanical support [8, 17]. We have previously shown that skeletal muscle rSO_2_ can predict adequacy of flow (i.e., mixed venous oxygenation) in patients with shock with preserved oxygen extraction; it can also be used to track effects of therapy [8, 18]. Despite new NIRS technologies and design of probes, skeletal muscle rSO_2_ monitoring remains technically more reliably compared to brain rSO_2_, because skeletal muscle is covered with a thin layer of skin and subcutis compared to brain, which is hidden in the skull and floating in the cerebrospinal fluid [19]. A short paper already reported an illustrative case series of skeletal muscle rSO_2_ use in five patients in the emergency department, showing fast response of skeletal muscle rSO_2_ value to loss or return of pulse [15].

Aim of our study is to test feasibility to monitor skeletal muscle rSO_2_ during CPR after OHCA and to assess changes of skeletal muscle rSO_2_ during CPR and after ROSC. In addition, we want to explore the relationship between skeletal muscle and brain rSO_2_.

## Methods

### Study design and setting

The single-center, prospective, non-randomized and observational study was conducted at a prehospital area that is covered by the Emergency Unit of Community Health Centre Ljubljana and the Rescue station of University Medical Centre Ljubljana during September 2019 and May 2022. The prehospital area has 1670 km^2^ and provides emergency services for around 450.000 inhabitants and additionally over 60.000 daily working migrants.

The research protocol received approval by Slovenian Medical Ethics Committee (No. 0120-334/2019/3); patients’ consent was waived because of the observational nature of the study and emergency setting. Study protocol was registered at clinicaltrials.gov (NCT04058925).

### Study intervention

All patients with non-traumatic cardiac arrest aged 18 or more were eligible for inclusion. Excluded patient were as follows: age below 18 years, pregnant women, traumatic cardiac arrest, hypothermic patient, drowned patient, patient who had additional extracorporeal CPR, patients who had achieved ROSC before the placement of NIRS device probes on the skin and if it was not possible to place NIRS probes on the patient within 5 min after start of ALS algorithm. Citeria for additional extracorporeal CPR are (all must be fulfilled): age < 55 years, witnessed cardiac arrest, appropriate BLS before ALS, primary shockable rhythm, unsuccessful advance life support resuscitation for at least 30 min (i.e., sustained/resistant ventricular fibrillation), estimated time to implantation of extracorporeal device less than 60 min from the time of collapse.

The team of doctor and two medical rescuers were dispatched by a health dispatcher after receiving information of a patient not showing signs of life. The doctor led resuscitation according to European Resuscitation Council guidelines for Advanced Life Support (ALS) [20, 21]. Immediately upon arrival, the prehospital team started with the ALS algorithm.

### Tissue oxygenation measurement

As soon as possible, one of the team members placed NIRS probes on the patient. NIRS device (SenSmart Model X-100, Nonin Medical, Inc. Playmouth, Minnesota, USA), which records rSO_2_ every 4 s, and disposable self-adhesive probes (SenSmart Nonin Medical, Inc. Playmouth, Minnesota, USA) were used. Each probe was marked with color and always placed on the same part of the body: blue probe for brain and yellow for skeletal muscle. The blue probe for measuring brain rSO_2_ was placed on the patient’s right side of forehead and the yellow probe to the patient’s right hand thenar to measure skeletal muscle rSO_2_. The patient’s thenar was used due to our previous experimental and clinical experience [8]. The probes were additionally fixed with medical grade adhesive tape to avoid discontinuation of measurements. The NIRS device screen was not covered, so the team members could fix probe position in case of bad contact. As consequence this study was unblinded. However, teams had instructions that measured rSO_2_ values must not influence decisions made by the resuscitation team regarding termination of resuscitation or continuing one. The measurement stopped when the patient was admitted to the Emergency department or when the doctor declared death of the patient and CPR was terminated [22]. Return of spontaneous circulation (ROSC) was defined as return of spontaneous palpable pulse and or breathing, coughing, movement of the patient, rise of etCO_2_ for more than 30 s [22].

**Fig. 1:**
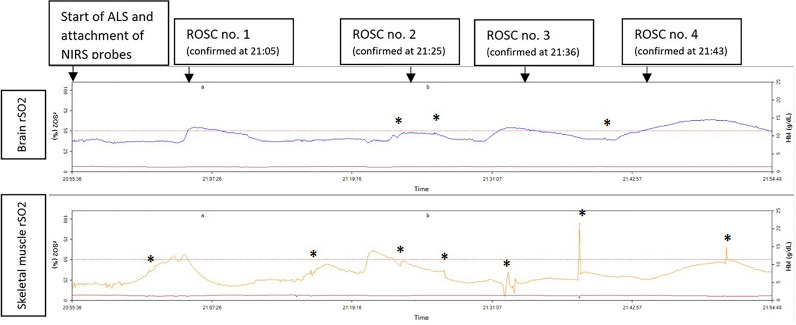
Graph example from one of CPRs with several ROSC (* marks artefact)

After intervention data were downloaded from the NIRS device by especially dedicated software (SenSmart, Version 1.0.1.0, Nonin Medical Inc., Minneapolis, MN USA) and paired with information from the intervention protocol. One of the graphs with measurements is presented in Fig. 1. Basal rSO_2_ was defined as an average of 4 measurements (average of rSO_2_ values in 16 s) of brain and skeletal muscle rSO_2_ after signal stabilization (approximately 12–16 s) after NIRS probes placement. We also recorded maximal rSO_2_ value during the CPR, difference between maximal–minimal rSO_2_ value (delta-rSO_2_), value of rSO_2_ at ROSC or the end of CPR with no-ROSC (end-CPR rSO_2_). rSO_2_ value of end-CPR rSO_2_ was average of 4 (16 s) measurements just before ROSC was confirmed or the resuscitation was terminated. Spiking signals, which were out of trend of the NIRS measurements, were considered as artefacts (Fig. 1) and we not included in analysis.

### Additional data

The following additional data were recorded: basic demographic data (age, gender), the time of call to emergency telephone number (112), the time of arrival of the emergency team on scene, the time of ROSC/time of death, duration of CPR, was cardiac arrest witnessed, were eyewitnesses doing BLS (basic life support), the first ECG rhythm, use of AED, intubation status, number of defibrillations, cumulative dose of used adrenaline, ECG rhythm at the end of CPR/intervention and 28-day survival.

### Primary outcome

Primary outcome was feasibility of skeletal muscle rSO_2_ measurement; how demanding is it to apply NIRS probes on two sites, what are the main problems of losing signal, how to fix the probes not to lose the signal.

### Secondary outcome

The secondary outcome was to find out if there are any changes in measured skeletal muscle rSO_2_ during CPR and before/after ROSC. We also want to test the relationship between basal skeletal muscle rSO_2_ and time to start ALS in patients with witnessed cardiac arrest and without BLS. We also want to test relationship between skeletal muscle and brain rSO_2_.

Power analysis: no previously published data were available for specific NIRS device used in our study, that is why absolute difference of mean skeletal muscle rSO_2_ = 20% (SD 10%) between patients with ROSC and no-ROSC (ratio of sample size 1:3) was estimated in the first 10 recruited patients. For estimated error (Type I. error of 0.05, Type II. error of 0.20) total sample size of 16 patients (4 in ROSC, 12 in no-ROSC group) would be necessary.

### Statistical analysis

The study population was divided into 2 groups according to outcome: ROSC and no-ROSC group. Continuous data were summarized as median (25th–75th quartile) compared by Mann–Whitney test for independent and Wilcoxon test for paired samples. Non-continuous data were summarized as the count (percentage). Chi-square test was used to compare non-continuous data. Rank correlation with Spearman's coefficient (rho) was used to test relationships between variables. Linear and non-linear regression methods was also used to test relationship between variables. MedCalc^®^ ver. 20.104 (MedCalc Software Ltd) software was used for the statistical analysis. *P* value < 0.05 was regarded as statistically significant.

## Results

**Fig. 2:**
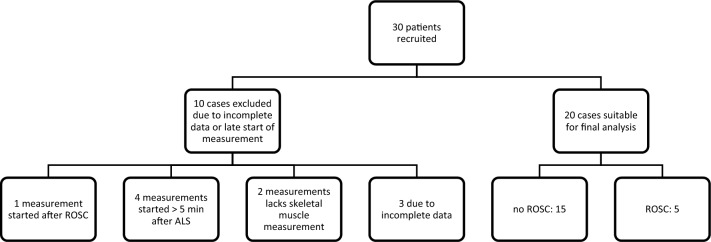
Flowchart of patients included in the study

Thirty patients were recruited. Ten patients were excluded due to different technical problems or violation of study protocol (Fig. 2): two patients due to disconnection of NIRS probe and consequent loss of skeletal muscle rSO_2_ signal during CPR; in four patients the probes were applied more than 5 min after arrival to the patient; in one patient probes were applied after ROSC; three patients were excluded due to irregular rSO_2_ signals, which have not allowed to determine predefined checkpoints.

Twenty patients with skeletal muscle rSO_2_ data were finally analyzed. In these patients, one patient had only skeletal muscle and no brain rSO_2_ recordings. Both skeletal muscle and brain rSO_2_ were recorded in 19 patients.

Basic demographic data, data about arrest and CPR are presented in Table 1. Twenty patients [age: 66.0 years (60.5–79.5), 65% male] with OHCA (50% witnessed, 70% with BLS), time to advance life support (ALS) 13.5 min (11.0–19.0) were finally analyzed. Cumulative dose of adrenaline was higher in no-ROSC group.

**Table 1:** Basic demographic and cardiac arrest data

	All (*n = *20)	ROSC (*n = *5)	No ROSC (*n = *15)	Statistics *p*
Age (years), median (interquartile range)	66.0 (60.5–79.5)	54.0 (48.75–69.75)	67.0 (63.5–80.25)	0.1152
Gender, male, *n* (%)	13 (65%)	5 (100%)	8 (53%)	0.0648
Witnessed arrest, *n* (%)	10 (50%)	1 (20%)	9 (60%)	0.1311
Bystander BLS, *n* (%)	14 (70%)	4 (80%)	10 (67%)	0.5829
Time to ALS (min), median (interquartile range)	13.5 (11.0–19.0)	18 (9.5–21.0)	13 (11.25–16.75)	0.7263
Primary rhythm				
VF	7 (35%)	1 (20%)	6 (40%)	0.95
Asystole	10 (50%)	3 (60%)	7 (47%)	0.88
PEA	3 (15%)	1 (20%)	2 (13%)	0.69
Cumulative dose of adrenalin (mg), median (interquartile range)	4,5 (3.0–6.5)	2 (0.75–4.5)	5 (3.25–7.0)	0.0432
Number of defibrillators, median (interquartile range)	1 (0–3)	1 (0–2.25)	1 (0–3.75)	0.8186
Orotracheal intubation, median (interquartile range)	1 (1–2)	1 (1–2)	1 (1–2)	1.0
Duration of ALS (min), median (interquartile range)	30 (15–47.5)	11 (5.5–16)	34 (24–54.75)	0.0034
28-Day survival, number (%)	1 (5%)	1 (20%)	0 (0%)	0.08

BLS, basic life support; ALS, advanced life support; VF, ventricular fibrillation; PEA, pulseless electrical activity

**Fig. 3:**
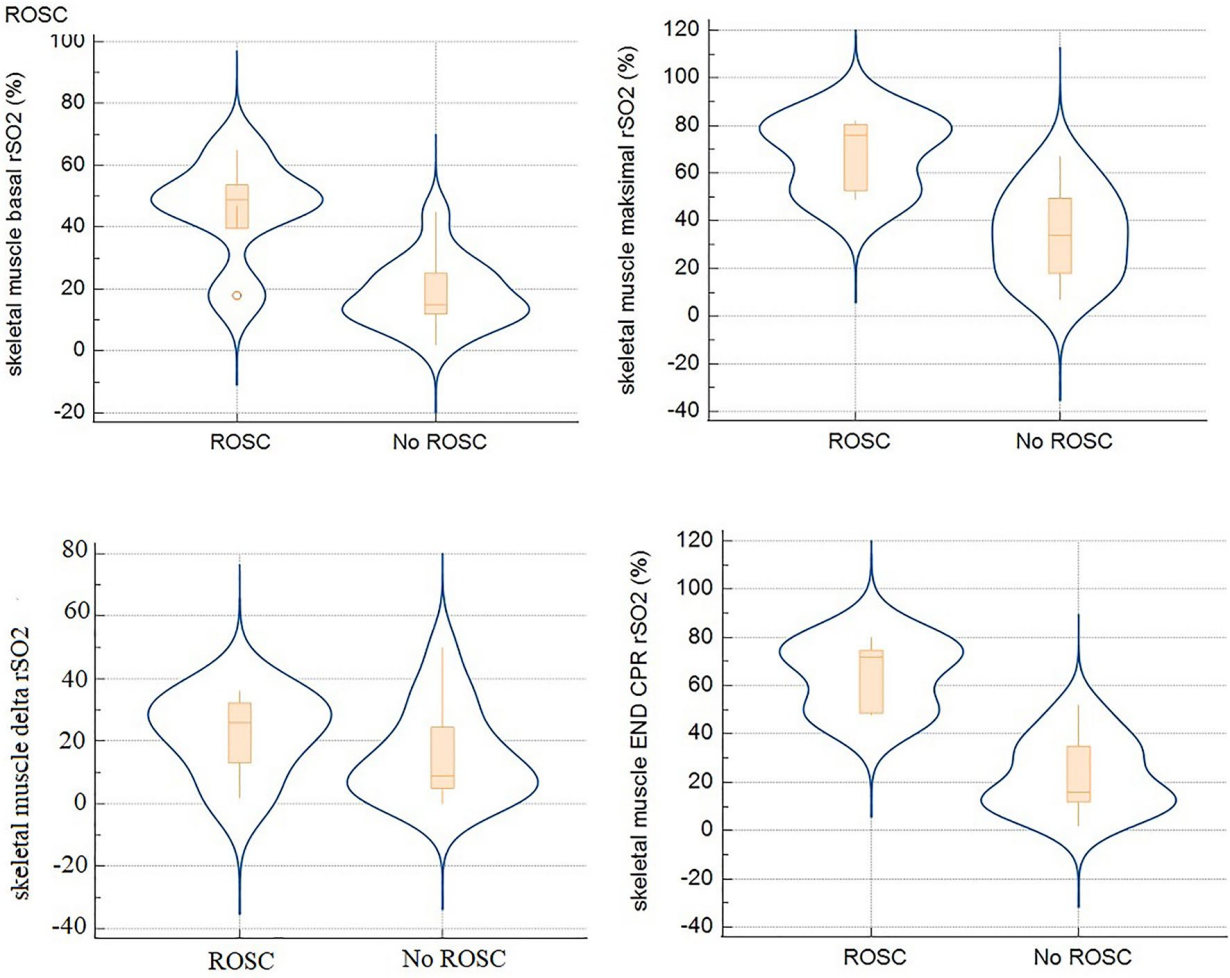
Skeletal muscle tissue oxygenation (rSO_2_) at the beginning, during and at the end of CPR. basal rSO_2_–rSO_2_ at the beginning of CPR, maximal rSO_2_—the highest rSO_2_ during CPR, delta RSO_2_—difference between maximal and minimal rSO_2_ during CPR, end-CPR rSO_2_—rSO_2_ at the end of CPR

Measurement of skeletal muscle rSO_2_ and brain rSO_2_ are presented in Table 2. There was no statistically significant difference between the basal skeletal muscle and the basal brain rSO_2_ (17.5 (12.8–26.0) vs. 31.0 (15.8–41.6), *P = * 0.1674, respectively). Basal, maximal and end-CPR skeletal muscle rSO_2_ were higher in ROSC compared to no-ROSC group (49.0% (39.7–53.7) vs. 15.0% (12.0–25.2), *P = * 0.006; 76.0% (52.7–80.5) vs. 34.0% (18.0–49.5), *P = * 0.005; 72.0% (48.7–74.7) vs. 16.0% (12.0–35.0), *P = * 0.002, respectively) (Fig. 3). Delta rSO_2_ for skeletal muscle was not significantly different in patients with ROSC and no-ROSC group.

**Table 2:** Skeletal muscle and brain regional tissue oxygenation during CPR

	Skeletal muscle				Brain			
	All (*n = *20)	ROSC (*n = *5)	No ROSC (*n = *15)	*p*	All (*n = *19)	ROSC (*n = *3)	No ROSC (*n = *16)	*p*
Basal rSO_2_ (%)	20.5 (14.0–32.0)	49.0 (39,7–53,7)	15.0 (12.0–25.2)	0.006	31.0 (15.8–41.6)	38.0	29.5 (14.5–42.5)	0.4
Maximal rSO_2_ (%)	44.0 (27.0–54.0)	76.0 (52.7–80.5)	34.0 (18.0–49.5)	0.005	45.0 (32.0–58.6)	77	42.0 (30.5–53.0)	0.01
Delta rSO_2_ (%)	15.5 (6.0–26.0)	26.0 (13.2–32.2)	9.0 (5.0–24,5)	0.3	14.0 (7.8–18.2)	27	10.5 (6.0–15.0)	0.007
End-CPR rSO_2_ (%)	31.0 (13.0–47.0)	72.0 (48.7–74.7)	16.0 (12.0–35.0)	0.002	47.5 (32.5–54.6)	77	39.0 (29.7–52.7)	0.01

Data are presented as median (interquartile range)

Basal rSO_2_, rSO_2_ at the beginning of CPR; Maximal rSO_2_, the highest rSO_2_ during CPR; Delta RSO_2_, difference between maximal and minimal rSO_2_ during CPR; end-CPR rSO_2_, rSO_2_ at the end of CPR

Basal brain rSO_2_ did not differ between ROSC and no-ROSC (38.0% vs. 29.5% (14.5–42.5), *P = * 0.4). Maximal, delta-rSO_2_ and end-CPR brain rSO_2_ were higher in ROSC compared to no-ROSC group (77% vs. 42.0% (30.5–53.0), *P = * 0.01; 27% vs. 10.5% (6.0–15.0), *P = * 0.007; 77% vs. 39.0% (29.7–52.7), *P = * 0.01, respectively) (Table 2).

**Fig. 4:**
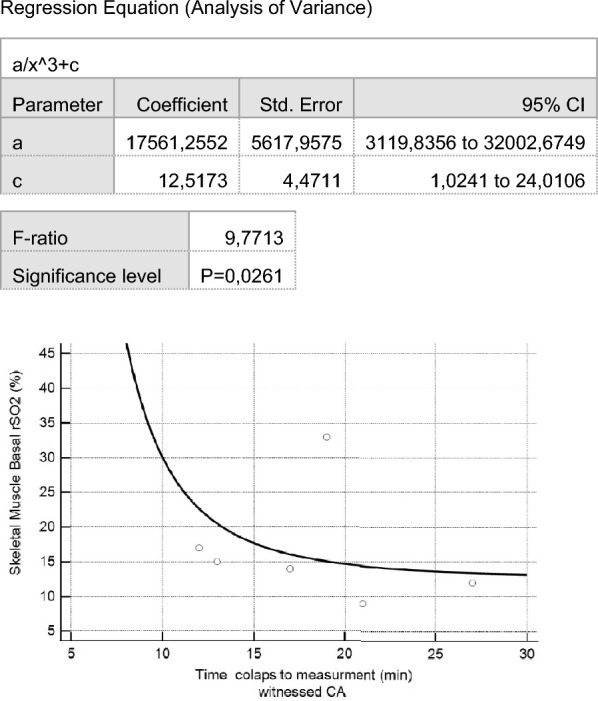
Relationship between time between collapse and Basal skeletal muscle rSO_2_ in witnessed cardiac arrest. Regression Equation (Analysis of Variance)

There was non-linear cubic relationship between duration of collapse to establishment of NIRS monitoring and basal skeletal muscle rSO_2_ in witnessed OHCA and without BLS (F-ratio = 9.7713, *P = * 0.0261) (Fig. 4).

**Fig. 5:**
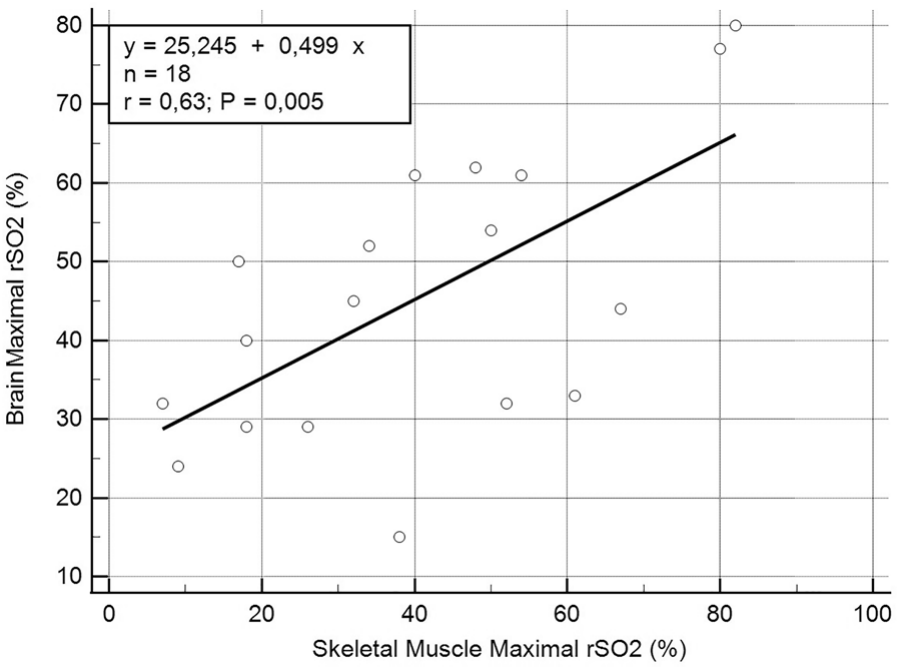
Correlation between Maximal skeletal muscle and brain rSO_2._ (*n = *18, Spearman's coefficient of rank correlation (rho): 0.578, *P = * 0.0121, 95% CI for rho: 0,152 to 0,823)

There was no correlation between basal rSO_2_, delta-rSO_2_ and end-CPR rSO_2_ of skeletal muscle and brain. There was a correlation between maximal skeletal muscle and brain rSO_2_ (*n = *18, rho: 0.578, *P = * 0.0121), which was confirmed also in linear regression model (*y* = 25.245 + 0.499 x, *r* = 0.63, *P = * 0.005) (Fig. 5).

## Discussion

The study confirmed the feasibility of monitoring skeletal muscle rSO_2_ in OHCA. Patients with ROSC had higher skeletal muscle rSO_2_ at the start of ALS (basal rSO_2_) and during CPR (maximal rSO_2_). There was a non-linear cubic relationship between basal rSO_2_ and time from collapse to start of rSO_2_ monitoring. There was also a linear relationship between the maximal values of skeletal muscle and brain rSO_2_.

During cardiac arrest there is decrease of rSO_2_, the value of decrease depends on the duration of no-flow/low flow and oxygen consumption of the tissue [8]. Basal skeletal muscle rSO_2_ is a surrogate for estimating the time of tissue low/no flow. Our study has shown a relationship between duration of witnessed cardiac arrest and basal skeletal muscle rSO_2_, which was, however, not significant due to low number of patients, because only patients with witnessed cardiac arrest and without BLS were include in that analysis. Basal skeletal muscle rSO_2_ were significantly different between ROSC and No ROSC groups, when all included patients were analyzed.

By rapid cuff inflation it is possible to stop flow through the arm, such as simulation of cardiac arrest, and evaluate skeletal muscle oxygen consumption. We have done this in patients with sepsis/septic shock and controls, aiming skeletal muscle rSO_2_ to decrease to 40% [23]. The rate of StO_2_ decrease during rapid cuff occlusion test was lower in septic shock patients compared to severe sepsis and controls (− 5 ± 2%/min vs. − 12 ± 2%/min vs. − 37 ± 7%/min, respectively; *P* < 0.001). In healthy volunteers we could measure rate of skeletal muscle rSO_2_ decrease during no-flow for longer period of time without any major risk, to explore skeletal muscle rSO_2_ kinetics and construct a normogram for estimating the duration of cardiac arrest for different age and gender groups.

In current study increase of skeletal muscle during resuscitation (delta rSO_2_) was not different between ROSC and No ROSC groups, this additionally emphasize the importance to start the resuscitation early as possible, when the basal skeletal muscle rSO_2_ (tissue oxygenation) is also still relatively high.

By additional fixation we have improved the position of NIRS probe on the thenar allowing more stable signal monitoring. The problem was big probe size, compared to thenar and higher possibility of detachment while manipulating patient’s hand. This fixation completely removed the possibility of losing contact and consequently signal during rSO_2_ monitoring even during different manipulations around and with the patient.

Despite the end of resuscitation (end-CPR rSO_2_) was different between ROSC and No-ROSC group, it is a very biased measure, since end-CPR by definition occurs much later in the no-ROSC group. This is a similar problem to the resuscitation-time bias seen in previous observational studies of the use of adrenaline during resuscitation from cardiac arrest [24–26]. End-CPR rSO2, furthermore, is an impracticable measure that cannot be used in a clinical relevant setting, because of the obvious fact that we cannot predict ROSC.

Brain rSO_2_ seems to be physiologically superior compared to other regional tissues. However, other rSO_2_, like kidney, are tested to give additional value to brain rSO_2_ during resuscitation in pediatric population [27, 28]. Monitoring of skeletal muscle rSO_2_ seem to be technically more reliably compared to brain rSO_2_, especially due to brain location in the body [19].

Several NIRS devices are available for clinical use. They differ according to numerous aspects, which include the algorithms adopted, the type of light source, the wavelengths of light emitted, the number and distance between the light emitters and detectors [19]. For example, INVOS system (5100C Cerebral/Somatic Oximeter; Medtronic, MN, USA) uses near-infrared light at two wavelengths [29]. Light travels from the light emitting diode of the sensor to either a proximal or distal detector, which allows separate data processing of shallow and deep optical signals. Data from the scalp and the surface tissue are subtracted and suppressed by spatial resolution, which reflects the rSO_2_ in deeper tissues [30]. The EQUANOX 7600 and also the SenSmart Model X-100 (Nonin Medical, MI, USA), the model that we have used, uses a dual light emitting and detecting sensor architecture, which has been shown to more effectively target the cerebral cortex and eliminate extracranial contamination from the scalp and skull. The Nonin system uses four wavelengths of near-infrared light. These added third and fourth wavelengths increase the accuracy of reporting the actual percent of oxygenated hemoglobin in the targeted tissues and can compensate for tissue factors that might otherwise reduce the accuracy of the measurements. This also allows the algorithm to reduce inter-subject variability, regardless of age, weight or skin color [30, 31]. We have previously shown high grade diversity of brain rSO_2_, with different NIRS devices, in patients with alkaptonuria, who had widespread tissue deposition of black pigment [19, 32, 33]. To guide our resuscitation efforts, we should probably not focus on only one modality, i.e., brain rSO_2_. Especially, because there is a report when good neurological outcome was achieved after prolonged CPR despite very low brain rSO_2_ [34].

Skeletal muscle rSO_2_ could also guide post-resuscitation care [17]. Continuous monitoring skeletal muscle rSO_2_ is already used in trauma patients and identifies the severity of shock [35]. Skeletal muscle rSO_2_ can track changes of systemic oxygen delivery during and after resuscitation of trauma patients or in patients heart failure patients/cardiogenic shock, who have preserved oxygen extraction [36]. New methods, such as near-infrared spectroscopy, which measures venous oxygen saturation in tissue from the near-infrared spectrum of the amplitude of respiration-induced absorption oscillations, may lead to the design of a non-invasive optical instrument capable of providing simultaneous and real-time measurements of local arterial, tissue and venous oxygen saturation.[37].

There was no statistically significant difference between the basal skeletal muscle and the basal brain rSO_2_, despite we would expect lower basal brain rSO_2_ due to higher cerebral oxygen consumption in normal human subjects compared to resting skeletal muscle oxygen consumption [38, 39]. There was also a very wide spread of basal rSO_2_. In pre-arrest state the patient could have centralization of flow to vital organ, and skeletal muscle rSO_2_ would be already low before cardiac arrest, as we previously have shown in patients with cardiogenic shock [8]. In our study, during resuscitation, there was lineal correlation between the maximal brain and skeletal muscle rSO_2_.

Repeatability of skeletal muscle rSO_2_ with NIRS during vascular occlusion test was confirmed in previous studies [40].

## Limitations

Current study has at least four major limitations. First, the number of recruited patients is low despite long recruiting period. The main cause is the SARS-CoV-2 epidemics, during which the study was temporally stopped to minimize workload of staff in protective clothing. Second, the study was only single center study. Our data should be confirmed in a bigger prospective multicenter study. Third, low number of patients with ROSC, did not allow to study time change of skeletal muscle rSO_2_ during resuscitation and prognostic value of skeletal muscle rSO_2_ for good neurological outcome. The fourth, study was not designed to study use of skeletal muscle rSO_2_ as post-resuscitation therapy guide and non-invasive estimation of adequacy of flow. It should be done in other multicenter study.

## Conclusions

Recording of skeletal muscle rSO_2_ during CPR in patients with OHCA is feasible. Basal and maximal skeletal muscle rSO_2_ were higher in ROSC compared to no-ROSC group. Skeletal muscle rSO_2_ during cardiac arrest could provide additional data to brain rSO_2_ on duration of arrest and efficiency of resuscitation efforts.

## Data Availability

The data sets used and/or analyzed during the current study are available from the corresponding author on reasonable request.

## Abbreviations

NIRS: Near-infrared spectroscopy
OHCA: Out of hospital cardiac arrest
ROSC: Return of spontaneous circulation
CPR: Cardiopulmonary resuscitation
BLS: Basic life support
ALS: Advanced life support
rSO_2_: Regional tissue oxygen saturation
