# Correction: Skeletal muscle oxygenation during cardiopulmonary resuscitation as a predictor of return of spontaneous circulation: a pilot study

**DOI:** 10.1186/s40001-023-01517-5

**Published:** 2023-11-16

**Authors:** Miha Košir, Hugon Možina, Matej Podbregar

**Affiliations:** 1 Faculty of Medicine, University of Ljubljana, Vrazov Trg 2, 1000 Ljubljana, Slovenia; 2 Unit SNMP, Community Health Centre Ljubljana, Bohoričeva Ulica 4, 1000 Ljubljana, Slovenia; 3 Emergency Department, University Medical Center Ljubljana, Zaloška Cesta 4, 1000 Ljubljana, Slovenia; 4 Department for Internal Intensive Care, General Hospital Celje, Oblakova Ulica 5, 3000 Celje, Slovenia

**Correction: European Journal of Medical Research (2023) 28:418** 10.1186/s40001-023-01393-z

In the original version of this article, the given and family names of all the authors were swapped and published incorrectly as Košir Miha, Možina Hugon and Podbregar Matej. The corrected author names should read as Miha Košir, Hugon Možina and Matej Podbregar. The original article [1] has been corrected.
